# Functional complaints and quality of life after transanal total mesorectal excision: a meta‐analysis

**DOI:** 10.1002/bjs.11566

**Published:** 2020-03-10

**Authors:** J. A. G. van der Heijden, T. Koëter, L. J. H. Smits, C. Sietses, J. B. Tuynman, A. J. G. Maaskant‐Braat, B. R. Klarenbeek, J. H. W. de Wilt

**Affiliations:** ^1^ Department of Surgery Radboud University Medical Centre Nijmegen Netherlands; ^2^ Department of Surgery Gelderse Vallei Hospital Ede Netherlands; ^3^ Department of Surgery Amsterdam UMC, Location VUmc Amsterdam Netherlands; ^4^ Department of Surgery Maxima Medical Centre Veldhoven Netherlands

## Abstract

**Background:**

Total mesorectal excision (TME) gives excellent oncological results in rectal cancer treatment, but patients may experience functional problems. A novel approach to performing TME is by single‐port transanal minimally invasive surgery. This systematic review evaluated the functional outcomes and quality of life after transanal and laparoscopic TME.

**Methods:**

A comprehensive search in PubMed, the Cochrane Library, Embase and the trial registers was conducted in May 2019. PRISMA guidelines were used. Data for meta‐analysis were pooled using a random‐effects model.

**Results:**

A total of 11 660 studies were identified, from which 14 studies and six conference abstracts involving 846 patients (599 transanal TME, 247 laparoscopic TME) were included. A substantial number of patients experienced functional problems consistent with low anterior resection syndrome (LARS). Meta‐analysis found no significant difference in major LARS between the two approaches (risk ratio 1·13, 95 per cent c.i. 0·94 to 1·35; *P* = 0·18). However, major heterogeneity was present in the studies together with poor reporting of functional baseline assessment.

**Conclusion:**

No differences in function were observed between transanal and laparoscopic TME.

## Graphical abstract

A novel surgical technique for total mesorectal excision (TME) is single‐port transanal minimally invasive surgery. This systematic review aimed to evaluate the functional outcomes and quality of life after transanal total mesorectal excision (TaTME) and compare them with those of laparoscopic TME (LapTME). No differences between TaTME and LapTME were seen in terms of impaired functioning and reduced quality of life after rectal cancer surgery. However, major heterogeneity was present in the studies together with poor reporting of functional baseline assessment.

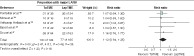

Comparable function

## Abstract (es)

**Antecedentes:**

La escisión total del mesorrecto (*total mesorectal excision*, TME) proporciona excelentes resultados oncológicos en el tratamiento del cáncer de recto, pero los pacientes pueden presentar trastornos funcionales. Un abordaje novedoso para realizar la TME es mediante cirugía transanal mínimamente invasiva de puerto único. En esta revisión sistemática se evaluaron los resultados funcionales y la calidad de vida después de TME transanal (TaTME) y TME laparoscópica (LapTME).

**Métodos:**

En mayo de 2019 se realizó una búsqueda exhaustiva en las bases de datos de Pubmed, Biblioteca Cochrane, EMBASE y en los registros de ensayos clínicos. Se utilizaron las guías PRISMA. Los datos para el metaanálisis se agruparon utilizando un modelo de efectos aleatorios.

**Resultados:**

Se identificaron un total de 11.660 estudios, de los cuales se incluyeron 14 estudios y 6 resúmenes de congresos con 846 pacientes (599 TaTME/247 LapTME). Un número sustancial de pacientes presentó trastornos funcionales consistentes con el síndrome de resección anterior baja (*low anterior resection syndrome*, LARS). El metaanálisis no encontró diferencias significativas en los porcentajes de LARS grave entre los dos abordajes (razón de oportunidades, *odds ratio*, OR 1,13; i.c. del 95% 0,94‐1,35; *P* = 0,18). Sin embargo, los estudios globalmente presentaron una gran heterogeneidad, así como una deficiente información sobre la evaluación funcional basal.

**Conclusión:**

No se observaron diferencias en la función entre TaTME y LapTME.

## Introduction

Total mesorectal excision (TME) is the standard surgical treatment for rectal cancer, with excellent long‐term local recurrence‐free and overall survival rates^1^. Over time, advances in technology led to a shift from open to laparoscopic surgery owing to favourable short‐term outcomes such as less pain, reduced blood loss and improved recovery time^2^, ^3^, ^4^, ^5^, ^6^. However, quality of life (QoL) and functional outcomes were not significantly improved by the laparoscopic approach^7^, ^8^. The latest developments are the robotic and the transanal approach. The latter, called transanal TME (TaTME) has been developed to overcome surgical difficulties experienced during distal pelvic dissection, especially in men with a narrow pelvis, a low tumour and a high BMI^9^. Long‐term results of randomized studies are awaited, especially since the Norwegian moratorium on TaTME owing to an unexpectedly high local recurrence rate^10^.

Although many studies have investigated functional bowel dysfunction after laparoscopic low anterior resection^11^, ^12^, ^13^, little is known about these functional sequelae after TaTME and their impact on QoL. The most common postoperative complaints, such as incontinence, urgency and frequent bowel movement, are described as low anterior resection syndrome (LARS). This syndrome has a severe adverse effect on QoL^14^, ^15^, ^16^. Known risk factors for the development of LARS are a low level of anastomosis, poor preoperative function and neoadjuvant chemoradiotherapy^17^, ^18^, ^19^, ^20^. With the TaTME technique, surgeons might choose a lower anastomosis for technical rather than oncological reasons, and urethral injuries are more likely^21^. Concerns regarding functional outcomes after TaTME have been expressed. This meta‐analysis was conducted to compare functional outcomes and QoL after TaTME and laparoscopic TME (LapTME).

## Methods

This review was conducted in accordance with PRISMA guidelines^22^, ^23^, with an *a priori* developed review protocol (PROSPERO; CRD42019126975). A comprehensive search was undertaken in PubMed, Embase, the Cochrane database and the trial registers. The full search strategy is available in *Appendix* *S1* (supporting information).

Two reviewers performed the selection process and reviewed all included studies. Discrepancies were resolved through discussion. The following inclusion criteria were applied: patients with rectal cancer who underwent TaTME and received any assessment of functional outcome or QoL. If a study also included patients who underwent LapTME, this group was used as a comparator for the TaTME group. All study designs with a population of ten or more patients were included. No filters for language or date were used. Studies were excluded if they evidently contained the same data, or were letters to the editor or expert opinions. If reported, the time from ileostomy closure to the evaluation of functional outcome was included. Quality assessment was performed by using the Newcastle–Ottawa Scale for observational studies^24^ and the Cochrane quality assessment tool for randomized trials^25^.

### Analysis

Basic descriptive statistics were used to summarize patient characteristics and outcome data. A meta‐analysis was performed if sufficient studies and adequate data were available. The Mantel–Haenszel method was used for dichotomous data. A random‐effects model was used and checked using a fixed‐effect model. If the requested data were not available, mean(s.d.) values were calculated for overall analysis, if possible^26^. A meta‐analysis of *P* values was performed in comparative studies of QoL data evaluated by the European Organization for Research and Treatment of Cancer (EORTC) questionnaires^27^. The Cochrane handbook 6 was used as a guideline for this analysis^28^. No funnel plots were presented, owing to the limited number of studies available for meta‐analysis^28^. Analyses were performed using Review Manager version 5.3.5 (Nordic Cochrane Centre, The Cochrane Collaboration, Copenhagen, Denmark), and Microsoft Excel® (Microsoft, Redmond, Washington, USA) for the meta‐analysis that combined *P* values.

## Results

### Study selection

**Figure 1:**
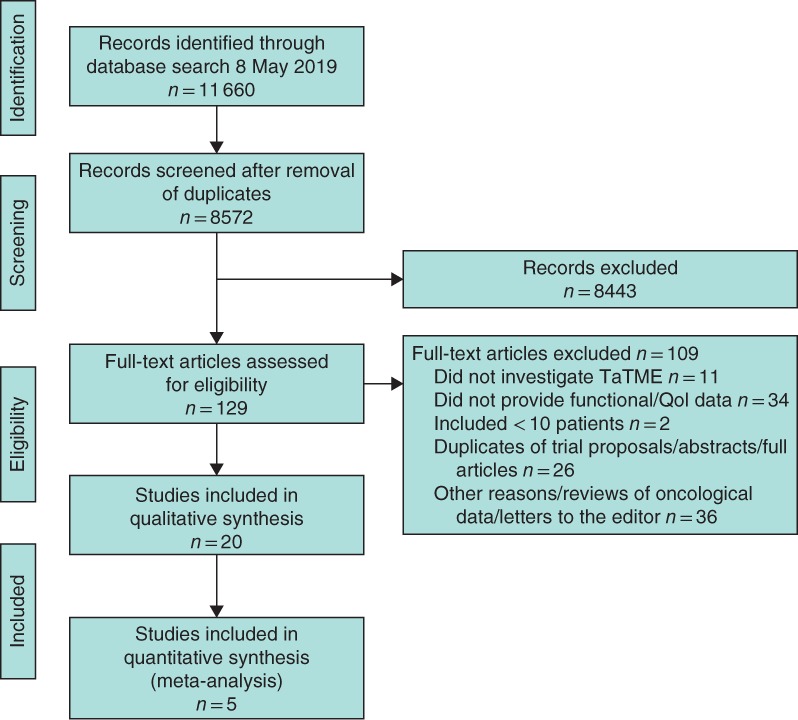
PRISMA diagram showing selection of articles for review TaTME, transanal total mesorectal excision; QoL, quality of life.

The search was performed in May 2019 and returned 11 660 articles after removal of duplicates from which left 8572 studies. After exclusion of irrelevant articles, 90 potentially relevant studies and 39 potentially relevant trials were assessed further. Eventually 14 studies and six conference abstracts were included (*Fig*. 1)^9^, ^29^, ^30^, ^31^, ^32^, ^33^, ^34^, ^35^, ^36^, ^37^, ^38^, ^39^, ^40^, ^41^, ^42^, ^43^, ^44^, ^45^, ^46^, ^47^. Studies were excluded for the following reasons: did not investigate TaTME (11), did not provide functional/QoL data (34), included fewer than ten patients (2) or other reasons (62).

### Study characteristics and quality control

Six retrospective (3 cross‐sectional, 2 cohort, 1 case–control) and 14 prospective (11 cohort, 2 cross‐sectional, 1 RCT) studies were included (*Table* *S1*, supporting information). The studies included 599 patients who underwent TaTME. A total of 247 patients who underwent LapTME were identified as a control group to compare with patients who underwent TaTME. Duration of follow‐up after surgery varied from 3 to 75 months. Seven studies included a baseline measurement in the study design. In the majority of studies, the tumour was located in the lower and middle rectum (tumour height 3·7–7·1 cm). Mean temporary ileostomy rates were 92·2 per cent in the TaTME group compared with 88·1 per cent in the LapTME group. Some 61·5 per cent of the patients received neoadjuvant treatment before TaTME compared with 70·8 per cent before LapTME. The height of anastomosis was not reported systematically, but was significantly lower after TaTME in the study of Mosquera and colleagues^45^. Other comparative studies showed no relevant differences in tumour height or site (mid, low, high).

Four of the included studies were of high quality based on the Newcastle–Ottawa Scale, scoring at least 7 points (*Table* *S2*, supporting information). Overall quality was acceptable, except that baseline measurements were not frequently reported and relatively few studies presented a comparator LapTME group. The only RCT was of good quality, except for an unclear risk of selective reporting.

### Bowel dysfunction

**Figure 2:**
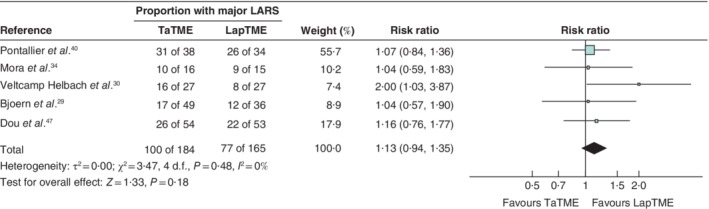
Meta‐analysis of the prevalence of major low anterior resection syndrome after transanal *versus* laparoscopic total mesorectal excision A Mantel–Haenszel random‐effects model was used for meta‐analysis. Risk ratios are shown with 95 per cent confidence intervals. The longest follow‐up data for each study were used. If a study favours laparoscopic total mesorectal excision (LapTME), fewer patients experienced major low anterior resection syndrome (LARS) in this group. TaTME, transanal total mesorectal excision.

Thirteen studies assessed bowel dysfunction by measuring the LARS score (*Table* 1), and five compared LARS scores after TaTME *versus* LapTME. Meta‐analysis showed no significant differences in the incidence of major LARS between the procedures (*Fig*. 2). Sensitivity analyses excluding studies with follow‐up of less than 12 months (risk ratio (RR) 1·15, 95 per cent c.i. 0·93 to 1·43) and studies with significant differences in baseline characteristics between TaTME and LapTME groups (RR 1·08, 0·89 to 1·32) showed no differences in bowel dysfunction outcomes between procedures.

**Table 1:** Bowel dysfunction as measured by low anterior resection score

Reference	No. of patients	Duration of follow‐up (months)	Total LARS score	No LARS	Minor LARS	Major LARS
Bjoern *et al*.^29^	49 TaTME	22·7 (10·3)*	26·2 (10·3)*	17 (35)	15 (31)	17 (35)
						
	36 LapTME	75·1 (17·6)*	20·6 (14·5)* (*P* = 0·054)	16 (44)	8 (22)	12 (33)
Veltcamp Helbach *et al*.^30^	27 TaTME	20·0 (6·6–44·4)†	27·7 (13·3)*	7 (26)	4 (15)	16 (59)
						
	27 LapTME	59·5 (39·7–82·0)†	24·0 (10·5)* (*P* = 0·267)	11 (41)	8 (30)	8 (30)
Turrado‐Rodriguez *et al*.^31^	80 TaTME	37·6‡	n.r.	31 (39)		49 (61)
Rubinkiewicz *et al*.^32^	25 TaTME	Baseline	5 (0–12)§	n.r.	n.r.	n.r.
						
		6	32 (30–37)§	0 (0)	4 (16)	21 (84)
Reali *et al*.^33^	29 TaTME	Baseline	n.r.	11 (38)	13 (45)	5 (17)
						
		24	n.r.	8 (28)	15 (52)	6 (21)
Mora *et al*.^34^	16 TaTME	6	n.r.	3 (19)	3 (19)	10 (63)
						
	15 LapTME	6	n.r.	4 (27)	2 (13)	9 (60)
						
Koedam *et al*.^37^	30 TaTME	Baseline	15·4 (7·3, 23·5)¶	16 (53)	10 (33)	4 (13)
						
		1	35·7 (32·9, 38·6)¶	0 (0)	6 (20)	24 (80)
						
		6	21·7 (13·6, 29·9)¶	14 (47)	6 (20)	10 (33)
Hanke *et al*.^38^	31 TaTME	3	25‡	n.r.	n.r.	n.r.
						
	17	6	21‡	n.r.	n.r.	n.r.
	13	9	18‡	n.r.	n.r.	n.r.
	10	12	10‡	n.r.	n.r.	n.r.
	7	18	10‡	n.r.	n.r.	n.r.
	4	24	2·5‡	n.r.	n.r.	n.r.
Pontallier *et al*.^40^	38 TaTME	>12	36 (12–42)†	n.r.	n.r.	31 (82)
						
	34 LapTME		37 (12–42)† (*P* = 0·977)	n.r.	n.r.	26 (76)
Kneist *et al*.^41^	10 TaTME	Baseline	n.r.	9 (90)	1 (10)	0 (0)
						
		3	28 (9–38)†	3 (30)	3 (30)	4 (40)
		6	26 (9–32)†	4 (40)	5 (50)	1 (10)
						
Keller *et al*.^44^	61 TaTME	Baseline	23·0 (9·7)*	22 (40)	20 (36)	13 (24)
						
		12	25·6 (8·0)*	n.r.	n.r.	n.r.
Leão *et al*.^46^	20 TaTME	1	32·7#	(14)	(7)	(79)
		3	n.r.	(23)	(8)	(69)
		6	n.r.	(38)	(23)	(38)
						
		12	19·5#	(50)	(40)	(10)
Dou *et al*.^47^	54 TaTME	17·2 (12·1–30·4)†	n.r.	n.r.	n.r.	26 (48)
						
	53 LapTME		n.r.	n.r.	n.r.	22 (42)

Values in parentheses are percentages unless indicated otherwise; values are

* mean(s.d.)

† median (range)

‡ median

§ median (i.q.r.)

¶ mean (95 per cent c.i.)

# mean. LARS, low anterior resection syndrome; TaTME, transanal total mesorectal excision

LapTME, laparoscopic total mesorectal excision; n.r., not reported. *P* values are shown for TaTME *versus* LapTME.

Bjoern and colleagues^29^ reported no significant difference in LARS scores after TaTME compared with LapTME (*P* = 0·054) (*Table* 1). For the subcategories clustering of stools (*P* = 0·017) and faecal urgency (*P* = 0·032), a significant disadvantage for TaTME was found. Koedam and co‐workers^37^ reported significantly worse LARS scores 1 month after TaTME surgery, but did not note a significant difference at 6 months compared with baseline scores. A significant increase in LARS scores was demonstrated after surgery in all studies^33^. However, these scores returned to baseline values in the majority of studies^33^, ^44^, ^46^.

### Continence

Eleven studies used the Wexner score to assess the level of continence (*Table* 2); two others^36^, ^44^ used the Vaizey or Kirwin score. All studies that performed a preoperative assessment of function^32^, ^39^, ^41^ confirmed that no major preoperative deviations in Wexner score were present. Summarizing data that reported Wexner scores at specific times (3, 6, 9, 12, 18, 24 months) showed a median Wexner score at 3 months of 9 (range 1–20)^38^, ^41^, ^43^. At 6 months, median scores ranged from 3 to 7^38^, ^41^, ^43^. Rouanet *et al*.^9^ recorded a median Wexner score of 11 after 12 months. Tuech and colleagues^42^ reported that three of 52 patients received a colostomy owing to faecal incontinence after a minimum of 12 months of conservative therapy. Three studies^40^, ^43^, ^45^ compared TaTME with LapTME and none of them reported significant differences.

**Table 2:** Continence status as measured by Wexner score

Reference	No. of patients	Duration of follow‐up (months)	Wexner score*	Wexner score > 10 (major incontinence)
Turrado‐Rodriguez *et al*.^31^	80 TaTME	37·6(17·7)†	10(5)†	n.r.
				
				
Rubinkiewicz *et al*.^32^	25 TaTME	Baseline	0 (0–2)	n.r.
				
		6	11 (8–12)	n.r.
Hanke *et al*.^38^	31 TaTME	3	9	n.r.
	17	6	6	n.r.
	13	9	4	n.r.
	10	12	2	n.r.
	7	18	4	n.r.
	4	24	0	n.r.
				
Elmore *et al*.^39^	12 TaTME	Baseline	n.r.	n.r.
				
		6	3 (1–8)	n.r.
Pontallier *et al*.^40^	38 TaTME	> 12	9 (2–20)	16 (42)
	34 LapTME		10 (3–20)(*P* = 0·932)	14 (41) (*P* = 0·936)
				
Kneist *et al*.^41^	10 TaTME	Baseline	1 (0–7)	0 (0)
				
		3	9 (1–20)	4 (40)
				
		6	7 (0–15)	3 (30)
Tuech *et al*.^42^	52 TEAP	> 12	4 (3–12)	7 (13) > 7 points
De'Angelis *et al*.^43^	32 TaTME	3	9 (3–15)	10 (32)
				
	32 LapTME	3	10·5 (4–19) (*P* = 0·115)	16 (50)
Rouanet *et al*.^9^	30 TEAP	12	11	n.r.
Keller *et al*.^44^	61 TaTME	Baseline	n.r.	n.r.
				
		12	n.r.	n.r.
Leão *et al*.^46^	20 TaTME	1	10·3	n.r.
	20	3	7·9	n.r.
	20	6	4·6	n.r.
	8	12	2·8	n.r.

Values in parentheses are percentages unless indicated otherwise

* values are median (range), except

† mean(s.d.). TaTME, transanal total mesorectal excision; n.r., not reported; LapTME, laparoscopic total mesorectal excision; TEAP, transanal endoscopic proctectomy. *P* values are shown for TaTME *versus* LapTME.

### Urogenital dysfunction

The International Index of Erectile Function (IIEF/IIEF‐5), International Prostate Symptom Score (IPPS) and Female Sexual Function Index (FSFI) were used to evaluate urogenital dysfunction after TaTME (*Table* 3).

**Table 3:** Urogenital dysfunction as measured by International Index of Erectile Function and International Prostate Symptom Score

Reference	No. of patients	Duration of follow‐up (months)	IIEF score	Patients with erectile dysfunction (IIEF score ≤ 21)	IPSS	IPSS quality‐of‐life score	IPSS category
Bjoern *et al*.^29^	37 TaTME	22·7(10·3)*	n.r.	n.r.	6·7(7·4)*	Score 1/2/3/4/5/6/7: 22/7/7/0/0/1/0	No: 6 (16)Mild: 17 (46)Moderate: 12 (32)Severe: 2 (5)
	20 LapTME	75·1(17·6)*	n.r.	n.r.	10·1(8·2)* (*P* = 0·060)	Score 1/2/3/4/5/6/7: 8/7/0/3/1/0/1 (*P* = 0·01)	No: 1 (5) Mild: 9 (45) Moderate: 8 (40) Severe: 2 (10) (*P* = 0·236)
							
Veltcamp Helbach *et al*.^30^	14 TaTME	20·0 (6·6–44·4)†	n.r.	n.r.	8(6·6)*	n.r.	No/mild: 7 (50) Moderate: 7 (50) Severe: 0 (0)
							
	18 LapTME	59·5 (39·7–82·0)†	n.r.	n.r.	6·7(6·3)* (*P* = 0·582)	n.r.	No/mild: 12 (67) Moderate: 5 (28) Severe: 1 (6) (*P* = 0·277)
Pontallier *et al*.^40^	21 TaTME	38 (15–39)† Functional assessment >12	17·5 (5–25)†	14 (67)	5·5 (0–23)†	1 (0–6)†	IPPS >10: 21%
							
							
							
	16 LapTME		7 (5–21)† (*P* = 0·119)	15 (93) (*P* = 0·108)	3·5 (0–27)† (*P* = 0·821)	1 (0–5)† (*P* = 0·967)	IPPS >10: 21% (*P* = 0·961)
Kneist *et al*.^41^	10 TaTME						
							
	9	Baseline	n.r.	n.r.	5 (0–31)†	1 (0–4)	No/mild: 6 (67) Moderate: 2 (22) Severe 1 (11)
							
	9	3	n.r.	n.r.	3 (1–20)†	n.r.	No/mild: 7 (78) Moderate: 1 (11) Severe: 1 (11)
							
	9	6	n.r.	n.r.	n.r.	n.r.	No/mild: 7 (78) Moderate: 1 (11) Severe: 1 (11)
							
	6	9	n.r.	n.r.	n.r.	n.r.	No/mild: 5 (83) Moderate: 1 (17) Severe: 0 (0)
Keller *et al*.^44^	TaTME 61						
	50	Baseline	19·3(5·9)*	n.r.	6·3(5·0)*	1·3(1·4)	n.r.
	50	12	17·6(6·4)*	n.r.	5·9(4·7)*	1·4(1·2)	n.r.

Values in parentheses are percentages unless indicated otherwise; values are

* mean(s.d.)

† median (range). The International Prostate Symptom Score (IPSS) ranges from 0 to 35, with categories no/mild (0–7), moderate (8–19) and severe (20–35) complaints. IIEF, International Index of Erectile Function; TaTME, transanal total mesorectal excision; n.r., not reported/not reported correctly; LapTME, laparoscopic total mesorectal excision. *P* values are shown for TaTME *versus* LapTME.

#### *Urogenital function in men*

Foo and colleagues^35^ noted that erectile function in 23 men worsened significantly after surgery (*P* = 0·002) but returned to baseline after 6 months (*P* = 0·142). Pontallier and co‐workers^40^ did not find any significant differences in IIEF scores (*P =* 0·119) or category of erectile dysfunction (IIEF 21 or less; *P* = 0·108). Regarding urological function, Foo *et al*.^35^ showed no significant differences in scores measured at baseline, and 3 and 6 months after surgery. In studies that compared TaTME with LapTME^29^, ^30^, ^40^, there were no significant difference in IPPS scores between procedures (*Table* 3). Bjoern and co‐workers^29^ reported a significant effect on the IPPS QoL score in favour of TaTME (*P* = 0·01).

#### *Urogenital function in women*

Pontallier and colleagues^40^ reported sexual dysfunction in two of five women after TaTME and in two of three in the LapTME group. Turrado‐Rodriguez and co‐workers^31^ reported sexual dysfunction in 17 of 26 women after TaTME and concluded that these outcomes were similar to those of LapTME.

### Quality‐of‐life assessment

Four different QoL questionnaires were used, namely the EuroQol Five Dimensions (EQ‐5D™; EuroQol Group, Rotterdam, the Netherlands), EORTC QLQ‐C30, QLQ‐CR29 and Faecal Incontinence Quality of Life scale (FIQL) questionnaire. The QLQ‐CR38 is also frequently used for colorectal cancer, but not in the studies included in the present review. EQ‐5D™ data are known to correlate weakly with changes in defaecation pattern^48^, and are shown in *Table* *S3* (supporting information).

#### *Faecal Incontinence Quality of Life scale*

Only one study^46^ included the FIQL, and reported baseline scores of 4·0 (lifestyle, coping/behaviour, embarrassment) and 4·4 (depression/self‐perception). A decrease in QoL scores was seen 1 and 3 months after surgery (lifestyle 2·1–2·4, coping 2·5–3·5, depression 2·2–2·5, embarrassment 2·0–3·2), but scores returned to baseline within 1 year after TaTME (lifestyle 3·8, other scores 3·9).

#### *EORTC QLQ‐C30*

Two studies presented QoL scores over time (*Table* *S4*, supporting information). Keller and colleagues^44^ reported that emotional function increased significantly after 1 year compared with preoperative measurements (*P* ≤ 0·01). Koedam and co‐workers^37^ described a significant decrease in QoL (*P* = 0·012), physical functioning (*P* = 0·001), role functioning (*P* = 0·001), fatigue (*P* = 0·002) and general pain (*P* = 0·001). After 6 months, these effects disappeared, except for social functioning (*P* = 0·013) and anal pain (*P* = 0·013), which remained significantly worse than at baseline.

Three studies^29^, ^30^, ^34^ compared TaTME with LapTME. Veltcamp Helbach and colleagues^30^ reported scores for role functioning (89·5 *versus* 80·2; *P =* 0·042), fatigue (12 *versus* 26·5; *P =* 0·021) and faecal incontinence (2·4 *versus* 14·8; *P =* 0·032) in favour of LapTME. A discrepancy between studies was found for the domain emotional functioning; scores favouring LapTME were reported by Bjoern *et al*.^29^ (83·51 *versus* 87·07; *P =* 0·041), whereas Mora and colleagues^34^ described better scores for TaTME (89·58 *versus* 77·38; *P =* 0·031). Functional scores for diarrhoea were in favour of LapTME in the study of Bjoern and co‐workers^29^ (17·69 *versus* 4·62; *P =* 0·009). In a meta‐analysis combining significance levels, no statistically significant differences were found between QoL subdomains for the comparative studies (*Table* *S4*, supporting information).

#### *EORTC QLQ‐CR29*

Buttock pain (*P =* 0·01) and faecal incontinence (*P =* 0·03) were significantly worse in the TaTME group^29^, ^30^. Scores on all other scales were comparable, including flatulence and sexual function. Mora *et al*.^34^ described more abdominal pain and a bloated feeling in the LapTME group. A meta‐analysis combining significance levels showed no significant differences between the QoL subdomains for the comparative studies (*Table* *S4*, supporting information).

## Discussion

The present review investigated the impact of TaTME on functional outcomes and QoL. A significant proportion of patients who underwent TaTME experienced impaired postoperative bowel function. These complaints appeared to be present equally in patients treated by transanal and laparoscopic approaches.

A potential advantage of TaTME is that it allows construction of a (low) anastomosis in patients in whom abdominoperineal resection would previously have been necessary^32^. However, since the introduction of TaTME, concerns have been raised about postoperative function and QoL owing to factors such as the low anastomosis, urethral injuries, insertion of the transanal platform and anal stretch^21^, ^49^. Anal stretch and dilatation carries a potential risk of damaging the sphincter complex during transanal surgery. Previous studies of transanal endoscopic microsurgery (TEM) showed that controlled anal dilatation caused significant decreases in resting and voluntary contraction pressures, but had no influence on Wexner scores indicating clinical incontinence^50^, or long‐term QoL after TEM^51^.

To the extent that the included studies allow, given their follow‐up and quality, TaTME appears to be similar to LapTME in terms of functional outcomes. Potential risk factors for functional outcomes after TaTME were not investigated in this review. In a meta‐analysis regarding major LARS, no significant differences were found between LapTME and TaTME (RR 1·13, 95 per cent c.i. 0·94 to 1·35). In several non‐comparative studies that analysed TaTME only, variations in outcomes were found that could be explained by patient characteristics. In the study of Bjoern and colleagues^29^, scores for the subcategories clustering of stools and faecal urgency reached statistical significance not in favour of TaTME, but it is important to note that this study failed to report several important patient characteristics (such as preoperative function) and showed a significant difference in the timing of questionnaires. Although LARS scores were impaired after TaTME, only a few patients were reported who underwent complete disconnection of the anastomosis and construction of colostomy owing to faecal incontinence^42^. Male erectile function worsened after surgery but returned to baseline within 6 months^35^. No differences in sexual function for women^31^ or urological function for men^29^, ^30^, ^40^ were described between the two approaches.

Discrepancies in results were found between studies that used the EORTC questionnaires to measure QoL. Emotional functioning scores favoured LapTME in the study by Bjoern and co‐workers^29^ but were reported to favour TaTME by Mora *et al*.^34^. A difference in follow‐up was suggested as an explanatory factor because median follow‐up was 22·7 months for TaTME but 75·1 months for LapTME in the Bjoern study. The duration of follow‐up was also suggested to explain the differences in individual domains described by Veltcamp Helbach *et al*.^30^ (role function, fatigue and faecal incontinence in favour of LapTME). Overall, QoL and global health status were comparable between the TaTME and LapTME groups. In terms of buttock pain^29^ and faecal incontinence^30^, QoL was worse after TaTME. It is remarkable that these QoL deteriorations were not detected by the functional assessment tools used in these studies.

Overall, reporting of the included studies was complete, except for the conference abstracts that were obviously restricted in reporting, and some did not report all QoL domains^34^. A wide variety of adequate and valid questionnaires were used to assess QoL and functional outcomes^52^, ^53^. The overall quality of evidence was moderate, owing to considerable heterogeneity, lack of baseline measurements and relatively small sample sizes. The heterogeneity may have been the result of wide selection criteria, but these were specifically chosen to allow review of all available functional TaTME data. Additional treatment, preoperative function, height of the tumour and anastomosis, and differences in follow‐up times were important factors contributing to heterogeneity and the interpretation of functional outcomes^20^. Height of anastomosis was not reported systematically, but was significantly lower among patients who underwent TaTME in the study of Mosquera and colleagues^45^. In other comparative studies, no relevant differences in tumour height (in centimetres) and site (mid, low, high) were found. Six of eight studies properly described the rate of neoadjuvant therapy, and generally patients in the TaTME group underwent neoadjuvant therapy less frequently, yet this difference was not statistically significant.

The main limitation of this study is the lack of large RCTs. The majority of the studies were heterogeneous comparative studies and only seven of 20 reported preoperative baseline measurements. In addition, the surgeon's learning curve was reported poorly^54^. These limitations make it difficult to reach firm conclusions. However, it is important to draw attention to the oncological concerns surrounding TaTME: an unexpected pattern of recurrences early after TaTME resulted in a moratorium in Norway^10^. Several studies^55^, ^56^, ^57^, ^58^, ^59^, ^60^, ^61^, ^62^, ^63^, ^64^, ^65^ are currently investigating different aspects of transanal methods of TME surgery. The COLOR III trial^66^ is comparing TaTME with LapTME in a large cohort that should provide decisive data about the safety of TaTME.

## Supporting information


**Appendix S1** Search strategy per database (Pubmed, EMBASE, Cochrane Library and the WHO and http://clinicaltrial.gov trial registers).

## References

[1] Brouwer NPM , Bos ACRK , Lemmens VEPP , Tanis PJ , Hugen N , Nagtegaal ID *et al* An overview of 25 years of incidence, treatment and outcome of colorectal cancer patients. Int J Cancer 2018; 143: 2758–2766. 30095162 10.1002/ijc.31785 PMC6282554

[2] Veldkamp R , Kuhry E , Hop WC , Jeekel J , Kazemier G , Bonjer HJ *et al.;* COlon cancer Laparoscopic or Open Resection Study Group (COLOR). Laparoscopic surgery *versus* open surgery for colon cancer: short‐term outcomes of a randomised trial. Lancet Oncol 2005; 6: 477–484. 15992696 10.1016/S1470-2045(05)70221-7

[3] Kang SB , Park JW , Jeong SY , Nam BH , Choi HS , Kim DW *et al* Open *versus* laparoscopic surgery for mid or low rectal cancer after neoadjuvant chemoradiotherapy (COREAN trial): short‐term outcomes of an open‐label randomised controlled trial. Lancet Oncol 2010; 11: 637–645. 20610322 10.1016/S1470-2045(10)70131-5

[4] Jeong SY , Park JW , Nam BH , Kim S , Kang SB , Lim SB *et al* Open *versus* laparoscopic surgery for mid‐rectal or low‐rectal cancer after neoadjuvant chemoradiotherapy (COREAN trial): survival outcomes of an open‐label, non‐inferiority, randomised controlled trial. Lancet Oncol 2014; 15: 767–774. 24837215 10.1016/S1470-2045(14)70205-0

[5] Jayne DG , Thorpe HC , Copeland J , Quirke P , Brown JM , Guillou PJ . Five‐year follow‐up of the Medical Research Council CLASICC trial of laparoscopically assisted *versus* open surgery for colorectal cancer. Br J Surg 2010; 97: 1638–1645. 20629110 10.1002/bjs.7160

[6] Bonjer HJ , Deijen CL , Abis GA , Cuesta MA , van der Pas MH , de Lange‐de Klerk ES *et al.;* COLOR II Study Group. A randomized trial of laparoscopic *versus* open surgery for rectal cancer. N Engl J Med 2015; 372: 1324–1332. 25830422 10.1056/NEJMoa1414882

[7] Andersson J , Abis G , Gellerstedt M , Angenete E , Angerås U , Cuesta MA *et al* Patient‐reported genitourinary dysfunction after laparoscopic and open rectal cancer surgery in a randomized trial (COLOR II). Br J Surg 2014; 101: 1272–1279. 24924798 10.1002/bjs.9550 PMC4282093

[8] Andersson J , Angenete E , Gellerstedt M , Angerås U , Jess P , Rosenberg J *et al* Health‐related quality of life after laparoscopic and open surgery for rectal cancer in a randomized trial. Br J Surg 2013; 100: 941–949. 23640671 10.1002/bjs.9144 PMC3672685

[9] Rouanet P , Mourregot A , Azar CC , Carrere S , Gutowski M , Quenet F *et al* Transanal endoscopic proctectomy: an innovative procedure for difficult resection of rectal tumors in men with narrow pelvis. Dis Colon Rectum 2013; 56: 408–415. 23478607 10.1097/DCR.0b013e3182756fa0

[10] Larsen SG , Pfeffer F , Korner H ; Norwegian Colorectal Cancer Group . Norwegian moratorium on transanal total mesorectal excision. Br J Surg 2019; 106: 1120–1121. 31304578 10.1002/bjs.11287

[11] Chen TY , Wiltink LM , Nout RA , Meershoek‐Klein Kranenbarg E , Laurberg S , Marijnen CA *et al* Bowel function 14 years after preoperative short‐course radiotherapy and total mesorectal excision for rectal cancer: report of a multicenter randomized trial. Clin Colorectal Cancer 2015; 14: 106–114. 25677122 10.1016/j.clcc.2014.12.007

[12] Emmertsen KJ , Laurberg S ; Rectal Cancer Function Study Group . Impact of bowel dysfunction on quality of life after sphincter‐preserving resection for rectal cancer. Br J Surg 2013; 100: 1377–1387. 23939851 10.1002/bjs.9223

[13] Dulskas A , Miliauskas P , Tikuisis R , Escalante R , Samalavicius NE . The functional results of radical rectal cancer surgery: review of the literature. Acta Chir Belg 2016; 116: 1–10. 27385133 10.1080/00015458.2015.1136482

[14] Vironen JH , Kairaluoma M , Aalto AM , Kellokumpu IH . Impact of functional results on quality of life after rectal cancer surgery. Dis Colon Rectum 2006; 49: 568–578. 16583289 10.1007/s10350-006-0513-6

[15] Bryant CL , Lunniss PJ , Knowles CH , Thaha MA , Chan CL . Anterior resection syndrome. Lancet Oncol 2012; 13: e403–e408. 22935240 10.1016/S1470-2045(12)70236-X

[16] Camilleri‐Brennan J , Ruta DA , Steele RJ . Patient generated index: new instrument for measuring quality of life in patients with rectal cancer. World J Surg 2002; 26: 1354–1359. 12297930 10.1007/s00268-002-6360-2

[17] Croese AD , Lonie JM , Trollope AF , Vangaveti VN , Ho YH . A meta‐analysis of the prevalence of low anterior resection syndrome and systematic review of risk factors. Int J Surg 2018; 56: 234–241. 29936195 10.1016/j.ijsu.2018.06.031

[18] Bregendahl S , Emmertsen KJ , Lous J , Laurberg S . Bowel dysfunction after low anterior resection with and without neoadjuvant therapy for rectal cancer: a population‐based cross‐sectional study. Colorectal Dis 2013; 15: 1130–1139. 23581977 10.1111/codi.12244

[19] Ekkarat P , Boonpipattanapong T , Tantiphlachiva K , Sangkhathat S . Factors determining low anterior resection syndrome after rectal cancer resection: a study in Thai patients. Asian J Surg 2016; 39: 225–231. 26340884 10.1016/j.asjsur.2015.07.003

[20] Mir SA , Chowdri NA , Parray FQ , Mir PA , Bashir Y , Nafae M . Sphincter‐saving surgeries for rectal cancer: a single center study from Kashmir. South Asian J Cancer 2013; 2: 227–231. 24455643 10.4103/2278-330X.119929 PMC3889046

[21] Sylla P , Knol JJ , D'Andrea AP , Perez RO , Atallah SB , Penna M *et al.;* International taTME Urethral Injury Collaborative. Urethral injury and other urologic injuries during transanal total mesorectal excision: an international collaborative study. Ann Surg 2019; [Epub ahead of print]. 10.1097/SLA.0000000000003597 31567502

[22] Panic N , Leoncini E , de Belvis G , Ricciardi W , Boccia S . Evaluation of the endorsement of the preferred reporting items for systematic reviews and meta‐analysis (PRISMA) statement on the quality of published systematic review and meta‐analyses. PLoS One 2013; 8: e83138. 24386151 10.1371/journal.pone.0083138 PMC3873291

[23] Moher D , Liberati A , Tetzlaff J , Altman DG ; PRISMA Group . Preferred reporting items for systematic reviews and meta‐analyses: the PRISMA statement. PLoS Med 2009; 6: e1000097. 19621072 10.1371/journal.pmed.1000097 PMC2707599

[24] Wells GA , Shea B , O'Connell D , Peterson J , Welch V , Losos M *et al* *The Newcastle–Ottawa Scale (NOS) for Assessing the Quality of Non‐Randomized Studies in Meta‐Analysis*; 2000 http://www.ohri.ca/programs/clinical_epidemiology/oxford.asp [accessed 4 June 2019].

[25] Sterne JAC , Savović J , Page MJ , Elbers RG , Blencowe NS , Boutron I *et al* RoB 2: a revised tool for assessing risk of bias in randomised trials. BMJ 2019; 366: l4898. 31462531 10.1136/bmj.l4898

[26] Hozo SP , Djulbegovic B , Hozo I . Estimating the mean and variance from the median, range, and the size of a sample. BMC Med Res Methodol 2005; 5: 13. 15840177 10.1186/1471-2288-5-13 PMC1097734

[27] Becker BJ . Combining significance levels In Handbook of Research Synthesis, Cooper H, Hedges L (eds). Russell Sage: New York, 1994; 215–230.

[28] Higgins JPT , Green S . Cochrane Handbook for Systematic Reviews of Interventions. John Wiley & Sons: Chichester, 2011.

[29] Bjoern MX , Nielsen S , Perdawood SK . Quality of life after surgery for rectal cancer: a comparison of functional outcomes after transanal and laparoscopic approaches. J Gastrointest Surg 2019; 23: 1623–1630. 30603861 10.1007/s11605-018-4057-6

[30] Veltcamp Helbach M , Koedam TWA , Knol JJ , Velthuis S , Bonjer HJ , Tuynman JB *et al* Quality of life after rectal cancer surgery: differences between laparoscopic and transanal total mesorectal excision. Surg Endosc 2019; 33: 79–87. 29967994 10.1007/s00464-018-6276-z PMC6336756

[31] Turrado‐Rodriguez V , Torroella AT , De Lacy Oliver F , Guarner Piquet P , Otero‐Pineiro A , Martin‐Perez B *et al* Functional outcomes after tatme: retrospective analysis of quality of life and pelvic function. Dis Colon Rectum 2018; 61: e222.

[32] Rubinkiewicz M , Zarzycki P , Czerwińska A , Wysocki M , Gajewska N , Torbicz G *et al* A quest for sphincter‐saving surgery in ultralow rectal tumours – a single‐centre cohort study. World J Surg Oncol 2018; 16: 218. 30404633 10.1186/s12957-018-1513-4 PMC6223085

[33] Reali C , Keller DS , Penna M , Hompes R . Low anterior resection syndrome: are we getting to the bottom of the problem with TaTME? Colorectal Dis 2018; 20(Suppl 4): 41.

[34] Mora L , Zarate A , Serra‐Aracil X , Pallisera A , Serra S , Navarro‐Soto S . [Functional impairment and quality of life after rectal cancer surgery.] Cir Cir 2018; 86: 140–147. 29809186 10.24875/CIRU.M18000022

[35] Foo CC , Tsang J , Lam W , Law WL , Lo O . A comparative study on the sexual and urinary functions after transanal total mesorectal excision and low anterior resection for rectal cancer. Colorectal Dis 2018; 20(Suppl 4): 35.28795776

[36] Lelong B , Meillat H , Zemmour C , Poizat F , Ewald J , Mege D *et al* Short‐ and mid‐term outcomes after endoscopic transanal or laparoscopic transabdominal total mesorectal excision for low rectal cancer: a single institutional case–control study. J Am Coll Surg 2017; 224: 917–925. 28024946 10.1016/j.jamcollsurg.2016.12.019

[37] Koedam TW , van Ramshorst GH , Deijen CL , Elfrink AK , Meijerink WJ , Bonjer HJ *et al* Transanal total mesorectal excision (TaTME) for rectal cancer: effects on patient‐reported quality of life and functional outcome. Tech Coloproctol 2017; 21: 25–33. 28044239 10.1007/s10151-016-1570-z PMC5285410

[38] Hanke LI , Kauff DW , Lang H , Kneist W . Ano(neo‐)rectal function after transanal total mesorectal excision (TaTME) for primary rectal cancer. Eur Surg Res 2017; 58(Suppl 1): 39.

[39] Elmore U , Vignali A , Cossu A , De Nardi P , Lemma M , Parise P *et al* Transanal total mesorectal excision for rectal cancer. Preliminary experience. Surg Endosc 2017; 31(Suppl 1): S364.

[40] Pontallier A , Denost Q , Van Geluwe B , Adam JP , Celerier B , Rullier E . Potential sexual function improvement by using transanal mesorectal approach for laparoscopic low rectal cancer excision. Surg Endosc 2016; 30: 4924–4933. 26944728 10.1007/s00464-016-4833-x

[41] Kneist W , Wachter N , Paschold M , Kauff DW , Rink AD , Lang H . Midterm functional results of taTME with neuromapping for low rectal cancer. Tech Coloproctol 2016; 20: 41–49. 26561031 10.1007/s10151-015-1390-6

[42] Tuech JJ , Karoui M , Bridoux V , Manceau G , Kianifard B , Michot FA . A step towards NOTES total mesorectal excision for rectal cancer: endoscopic trans anal proctectomy (ETAP). Surg Endosc 2013; 1: S28. 10.1097/SLA.0000000000000994 25361216

[43] de'Angelis N , Portigliotti L , Azoulay D , Brunetti F . Transanal total mesorectal excision for rectal cancer: a single center experience and systematic review of the literature. Langenbecks Arch Surg 2015; 400: 945–959. 26497544 10.1007/s00423-015-1350-7

[44] Keller DS , Reali C , Spinelli A , Penna M , Di Candido F , Cunningham C *et al* Patient‐reported functional and quality‐of‐life outcomes after transanal total mesorectal excision. Br J Surg 2019; 106: 364–366. 30714147 10.1002/bjs.11069

[45] Mosquera C , Licardie E , Bravo D , Madarro C , Rosales J , Romero JA *et al* Fecal incontinence after surgical treatment of middle–low rectal cancer. Laparoscopic low anterior resection *versus* tatme. Surg Endosc 2019; 33(Suppl 1): S281.

[46] Leão P , Santos C , Goulart A , Caetano AC , Sousa M , Hogemann G *et al* TaTME: analysis of the evacuatory outcomes and EUS anal sphincter. Minim Invasive Ther Allied Technol 2019; 28: 332–337. 30888248 10.1080/13645706.2019.1567555

[47] Dou R , Sun W , Luo S , Hou Y , Zhang C , Kang L . [Comparison of postoperative bowel function between patients undergoing transanal and laparoscopic total mesorectal excision.] Zhonghua Wei Chang Wai Ke Za Zhi 2019; 22: 246–254. 30919377

[48] Deutekom M , Terra MP , Dobben AC , Dijkgraaf MG , Felt‐Bersma RJ , Stoker J *et al* Selecting an outcome measure for evaluating treatment in fecal incontinence. Dis Colon Rectum 2005; 48: 2294–2301. 16400514 10.1007/s10350-005-0162-1

[49] Buchs NC , Nicholson GA , Ris F , Mortensen NJ , Hompes R . Transanal total mesorectal excision: a valid option for rectal cancer? World J Gastroenterol 2015; 21: 11 700–11 708. 10.3748/wjg.v21.i41.11700 PMC4631971 26556997

[50] Mora López L , Serra Aracil X , Hermoso Bosch J , Rebasa P , Navarro Soto S . Study of anorectal function after transanal endoscopic surgery. Int J Surg 2015; 13: 142–147. 25486265 10.1016/j.ijsu.2014.11.021

[51] D'Ambrosio G , Picchetto A , Campo S , Palma R , Panetta C , De Laurentis F *et al* Quality of life in patients with loco‐regional rectal cancer after ELRR by TEM *versus* VLS TME after nChRT: long‐term results. Surg Endosc 2019; 33: 941–948. 30421081 10.1007/s00464-018-6583-4

[52] Zerillo JA , Schouwenburg MG , van Bommel ACM , Stowell C , Lippa J , Bauer D *et al.;* Colorectal Cancer Working Group of the International Consortium for Health Outcomes Measurement (ICHOM). An international collaborative standardizing a comprehensive patient‐centered outcomes measurement set for colorectal cancer. JAMA Oncol 2017; 3: 686–694. 28384684 10.1001/jamaoncol.2017.0417

[53] COMET Initiative . *Core Outcome Measures in Effectiveness Trials* Search term: colorectal cancer. http://www.comet-initiative.org/ [accessed 2 June 2019].

[54] Koedam TWA , Veltcamp Helbach M , van de Ven PM , Kruyt PM , van Heek NT , Bonjer HJ *et al* Transanal total mesorectal excision for rectal cancer: evaluation of the learning curve. Tech Coloproctol 2018; 22: 279–287. 29569099 10.1007/s10151-018-1771-8

[55] ClinicalTrials.gov . *Rectal Surgery Evaluation Trial (RESET)* https://clinicaltrials.gov/show/NCT03574493 [accessed 2 June 2019].

[56] ClinicalTrials.gov . *Trans‐anal* Versus *Laparoscopic TME for Mid and Low Rectal Cancer (MansTaTME)* https://clinicaltrials.gov/show/NCT03242187 [accessed 2 June 2019].

[57] ClinicalTrials.gov . *Laparoscopic Assisted Transanal Resection of Rectal Cancer With Total Mesorectal Excision* https://clinicaltrials.gov/show/NCT03171298 [accessed 2 June 2019].

[58] ClinicalTrials.gov . *Transanal Minimal Invasive Surgery* Versus *Conventional Laparoscopic Surgery for Rectal Cancer* https://clinicaltrials.gov/show/NCT02252250 [accessed 2 June 2019].

[59] Serra‐Aracil X , Mora L , Pericay C , Delgado S , Targarona E , Vallribera F *et al* Phase III multicenter, prospective, controlled, randomized trial to evaluate the safety and efficacy of treatment of rectal cancer T2–T3S (superficial) N0, M0 with preoperative chemoradiotherapy and transanal endoscopic microsurgery *versus* total mesorectal excision. Preliminary results. Dis Colon Rectum 2015; 58: e323–e324.

[60] ClinicalTrials.gov . *Laparoscopic Assisted Transanal Total Mesorectal Excision for Rectal Cancer in Low Site (LATERAL‐01)* https://clinicaltrials.gov/show/NCT03253302 [accessed 2 June 2019].

[61] ClinicalTrials.gov . *Transanal Total Mesorectal Excision for Rectal Cancer on Anal Physiology + Fecal Incontinence* https://clinicaltrials.gov/ct2/show/NCT03283540 [accessed 2 June 2019].

[62] ClinicalTrials.gov . *Multicenter Phase II Study of Transanal TME (taTME)* https://clinicaltrials.gov/ct2/show/NCT03144765 [accessed 2 June 2019].

[63] ClinicalTrials.gov . *Physical Exercise for Colorectal Cancer Patients After Transanal Total Mesorectal Excision* https://clinicaltrials.gov/ct2/show/NCT03120104 [accessed 2 June 2019].

[64] Luo S , Wang J , Kang L , Chen W . Transanal *versus* laparoscopic total mesorectal excision for low rectal cancer: a multicenter randomized phase III clinical trial (TaLaR trial) protocol. J Clin Oncol 2017; 35(Suppl 1). 10.1093/gastro/goaa083 PMC7962745 33747528

[65] Lacy AM , Espin E , Biondo S , Fernandez‐Hevia M , Tasende M , Jimenez M *et al* Transanal total mesorectal excision *versus* laparoscopic total mesorectal excision: a randomized study comparing 30‐day postoperative morbidity. Colorectal Dis 2014; 3: 106.

[66] Deijen CL , Velthuis S , Tsai A , Mavroveli S , de Lange‐de Klerk ES , Sietses C *et al* COLOR III: a multicentre randomised clinical trial comparing transanal TME *versus* laparoscopic TME for mid and low rectal cancer. Surg Endosc 2016; 30: 3210–3215. 26537907 10.1007/s00464-015-4615-x PMC4956704

