# Supplementary material for: Functional complaints and quality of life after transanal total mesorectal excision: a meta‐analysis
Source: Br J Surg. 2020 Mar 10;107(5):489–98. doi: 10.1002/bjs.11566 (PMC7155085; doi:10.1002/bjs.11566)
Supplement: Supplementary file 1 — Appendix S1 Search strategy per database (Pubmed, EMBASE, Cochrane Library and the WHO and http://clinicaltrial.gov trial registers). [file BJS-107-489-s001.docx]

**BJS11566**

**Functional complaints and quality of life after transanal total mesorectal excision: a meta-analysis**

J. A. G. van der Heijden, T. Koëter, L. J. H. Smits, C. Sietses, J. B. Tuynman , A. J. G. Maaskant-Braat, B. R. Klarenbeek and J. H. W. de Wilt

**Appendix S1 Search strategy per database (Pubmed, EMBASE, Cochrane Library and the WHO and ClinicalTrial.gov trial registers).**

**PubMed search:**
(“rectal Neoplasms”[Mesh] OR ((“neoplasms”[mesh] OR neoplas*[tw] OR tumor*[tw] OR tumour*[tw] OR cancer*[tw] OR malignan*[tw] OR oncolog*[tw] OR carcinom*[tw] OR adenocarcinom*[tw]) AND (“Rectum”[Mesh] OR rectum[tiab] OR rectal[tiab] OR colorect*[tiab] OR mesorect*[tiab]))) AND ("Natural Orifice Endoscopic Surgery"[Mesh] OR TaTME[tiab] OR TME[tiab] OR TAMIS[tiab] OR NOTES[tiab] OR protectomy[tiab] OR ((transanal*[tiab] OR trans-anal*[tiab]) AND (excision*[tiab] OR resection*[tiab] OR “surgery”[Subheading] OR surgery[tiab] OR surgical[tiab] OR operati*[tiab] OR “Rectal Neoplasms/surgery”[Mesh:noexp])))

*Conducted on 16-01-2019, number of hits: 3468*

**EMBASE search:**

(exp rectum tumor/ OR ((exp neoplasm/ OR neoplas*.mp. OR tumor*.mp. OR tumour*.mp. OR cancer*.mp. OR malignan*.mp. OR oncolog*.mp. OR carcinom*.mp. OR adenocarcinom*.mp.) AND (exp rectum/ OR rectum.ti,ab,kw. OR rectal.ti,ab,kw. OR colorect*.ti,ab,kw. OR mesorect*.ti,ab,kw.))) AND (natural orifice transluminal endoscopic surgery/ OR TaTME.ti,ab,kw. OR TME.ti,ab,kw. OR TAMIS.ti,ab,kw. OR NOTES.ti,ab,kw. OR protectomy.ti,ab,kw. OR ((transanal*.ti,ab,kw. OR trans-anal*.ti,ab,kw.) AND (excision*.ti,ab,kw. OR resection*.ti,ab,kw. OR surgery.fs. OR surgery.ti,ab,kw. OR surgical.ti,ab,kw. OR operati*.ti,ab,kw. OR exp rectum tumor/su)))

*
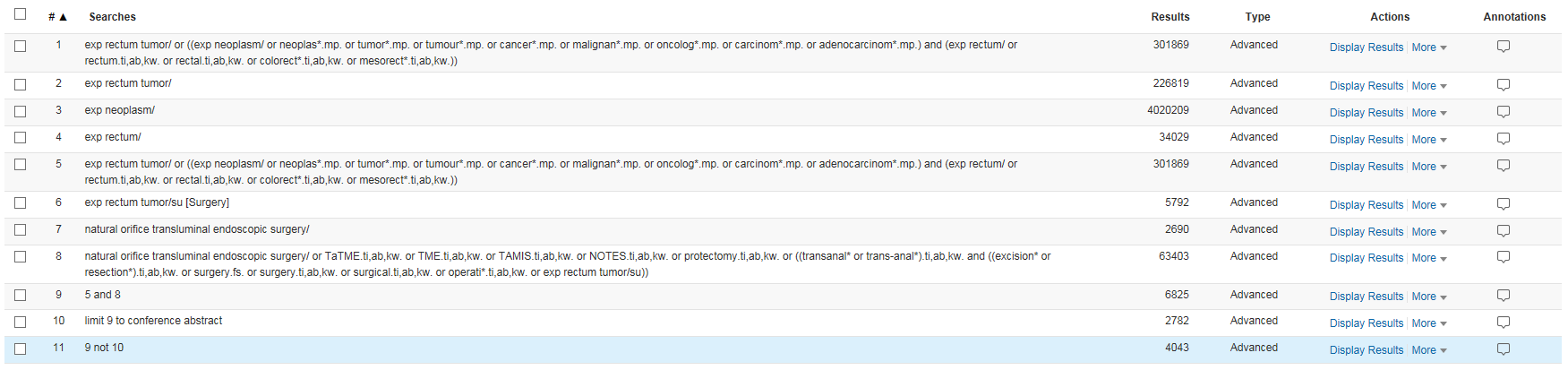
Conducted on 16-01-2019, number of hits: 6825. These hits include 2782 conference abstracts. These were screened separately, which explains why the included number of articles is 4043.*

**Cochrane search:**


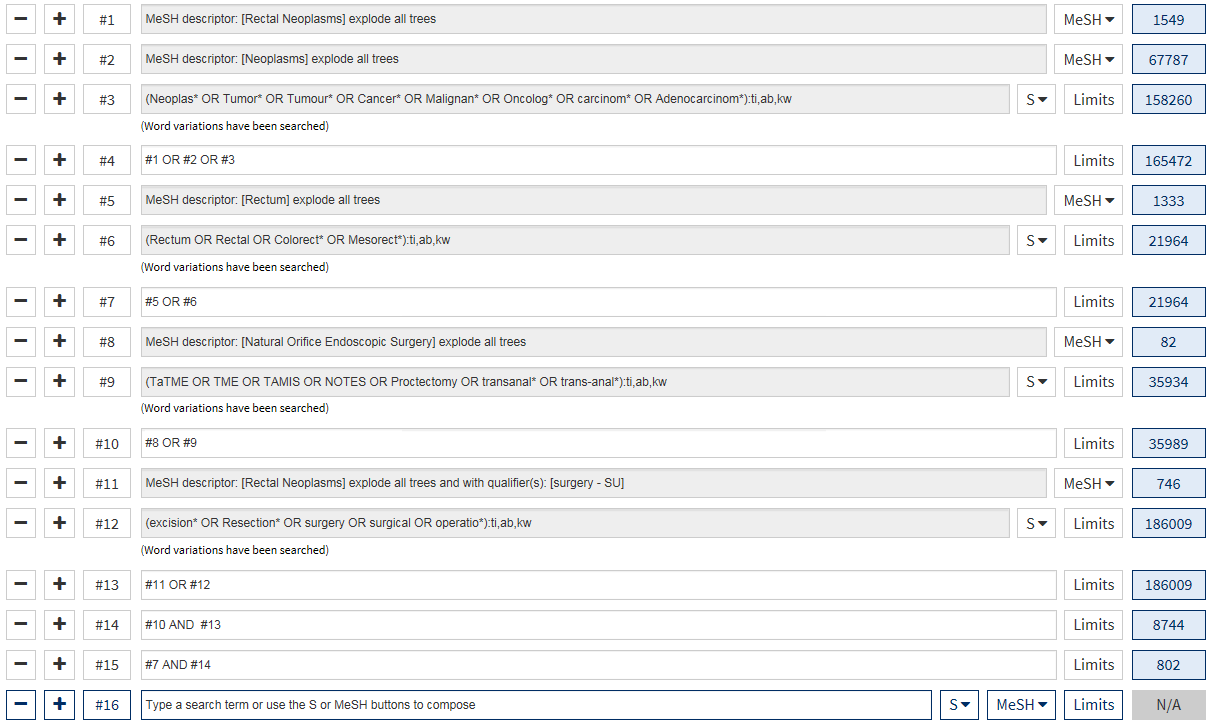


*Conducted on 16-01-2019, number of hits: 802.*

**Trial registers search terms:**

**ClinicalTrials.Gov**
Search terms: (Transanal total mesorectal excision OR TaTME OR TME OR NOTES) AND (rectal neoplasms OR rectal cancer)

*Search conducted on 16-01-2019, number of hits: 73.*

**WHO trial register**

Search terms: (Transanal total mesorectal excision OR TaTME) AND (rectal neoplasms OR rectal cancer OR rectal)

*Search conducted on 16-01-2019, number of hits: 12.*

**Updated literature search on 08-05-2019:**

Pubmed - number of hits since previous search 01-2019: 126

EMBASE - number of hits since 01-2019: 212 (of which 74 are conference abstracts).

Cochrane library - number of hits since 01-2019: 237

Total hits in the update: 575. After duplications removal 480 remain.

After title/abstract screening: 21 remaining of which eventually 3 were included for analysis.

**Table S1 Characteristics of included studies**

| **Study ID, country, type of literature** | **Year** | **TaTME or LapTME (n)** | **Follow-up, months (median, range) *** | **Sex (m/f)** | **Age, years**  **(mean, SD) *** | **Tumor height, cm (median, range) *** | **Tumor site (low/mid/**  **high)** | **ASA classification (I/II/III/IV/V)** | **Ileostomy**  **(n, %)** | **Time to reversal ileostomy (months)** |
| --- | --- | --- | --- | --- | --- | --- | --- | --- | --- | --- |
| #1 Bjoern2019, Denmark, cross-sectional, retrospective, full article. **^29^** | 2019 | 49 TaTME | 22.69 mean  SD 10.31 | 37/12 | 64.88 (9.65) | <6 cm: 4 | Mid | 21/20/8/0/0 | 49 (100%) | 3 |
|  |  | 36 LapTME | 75.08 mean  SD 17.61 | 16/20 | 62.42 (10.15) | <6 cm: 2 | Mid | 17/18/1/0/0 | 36 (100%) | 3 |
| #2 Veltcamp2018, Netherlands, cross-sectional, retrospective, full article. **^30^** | 2018 | 27 TaTME | 20.0 (6.6-44.4) | 18/9 | 68.0 (95%CI 64.4-71.6) | n.r. | low 9, mid 14, high 4 | 5/20/2/0/0 | 22 (81%) | 1.5 |
|  |  | 27 LapTME | 59.5  (39.7-82.0) | 20/7 | 62.7 (95%CI 59.6-65.7) | n.r. | low 7, mid 18, high 2 | 13/12/2/0/0 | 22 (81%) | 1.5 |
| #3 Turrado-  Rodriguez2018, Spain, cross-sectional, **^31^** retrospective, abstract. | 2018 | 80 TaTME | 37.6 median,  SD 17.7 | 54/26 | 65.7 (13.0) | n.r. | n.r. | n.r. | n.r. | n.r. |
| #4 Rubinkiewicz2018, Poland, cohort, retrospective, full article. **^32^** | 2018 | 25 TaTME | 6 | 19/6 | 65 median, IQR 59.5-72.5 | 2 (1-4) | low | 0/21/4/0/0 | 25 (100%) | n.r. |
| #5 Reali2018, U.K., cohort, prospective, abstract. **^33^** | 2018 | 29 TaTME | 24 | 25/4 | n.r. | 6.1 mean,  SD 2.1 | n.r. | n.r. | n.r. | n.r. |
| #6 Mora2018, Spain, cohort, prospective, full article. **^34^** | 2018 | 16 TaTME | >6 | 12/4 | 59.94 | 7.44 mean | n.r. | 0/11/4/1/0 | 16 (100%) | n.r. |
|  |  | 15 LapTME | >6 | 10/5 | 64.0 | 7.93 mean | n.r. | 0/9/6/0/0 | 15 (100%) | n.r. |
| #7 Foo2018, N.R., cohort, prospective, abstract. **^35^** | 2018 | 23 TaTME | 6 | 23/0 | n.r. | n.r. | n.r. | n.r. | n.r. | n.r. |
| #8 Lelong2017, France, case-control, full article **^36^** | 2017 | 34 TaTME | 31.9 | 23/11 | n.r. | n.r. | low | n.r. | 34 (100%) | n.r. |
|  |  | 38 LapTME | 31.9 | 22/16 | n.r. | n.r. | low | n.r. | 38 (100%) | n.r. |
| #9 Koedam2017, **^37^** Netherlands, cohort, prospective, full article. | 2017 | 30 TaTME | 6 | 21/9 | 66 median,  SD 9.3 | 6.0,  IQR 4.0-8.0 | n.r. | I-II: 27 (90%) | 24 (80%) | n.r. |
| #10 Hanke2017, Germany, cohort, prospective, abstract. **^38^** | 2017 | 31 TaTME | 24 | n.r. | 60 median. IQR 23-76 | n.r. | n.r. | n.r. | n.r. | n.r. |
| #11 Elmore2017, Italy, cohort, prospective, abstract. **^39^** | 2017 | 12 TaTME | 6 | n.r. | n.r. | n.r. | low/mid | n.r. | n.r. | n.r. |
| #12 Pontallier2016, France, RCT, full article. **^40^** | 2016 | 38 TaTME | 38 (13-59) | 26/12 | 62.0 median, range 39-81 | 4 (2-6) | Low | 28/15/0/0/0 | n.r. | n.r. |
|  |  | 34 LapTME | 38 (13-59) | 21/13 | 62 median, range 35-82 | 4 (2-6) | Low | 22/12/0/0/0 | n.r. | n.r. |
| #13 Kneist2016, **^41^** Germany, cohort, prospective, full article. | 2016 | 10 TaTME | 6 | 9/1 | 55.4 mean, range 42-65 | 4.6 mean,  range 4-5.5 | Low | 3/6/10/0/0 | 9 (90%) | 5 |
| #14 Tuech2015, France, cross-sectional, **^42^** prospective, full article. | 2015 | 56 TaTME | >12 | 41/15 | 65 median, range 39-83 | 4 (0-5) | low | 5/40/11/0/0 | 46 (82.1%) | 2-3 |
| #15 Angelis2015, **^43^** France, cross-sectional, prospective, full article. | 2015 | 32 TaTME | 3 | 21/11 | 64.91 (10.05) | 4 (2.5-5) | low | 21/10/1/0/0 | 32 (100%) | n.r. |
|  |  | 32 LapTME | 3 | 21/11 | 67.16 (9.61) | 3.7 (2.5-5) | low | 18/13/1/0/0 | 32 (100%) | n.r. |
| #16 Rouanet2013, **^9^** France, cohort, prospective, full article. | 2013 | 21 TaTME | >12 | 30/0 | 65 median, range 39-83 | n.r. | low 20, mid 10, high 0 | n.r. | n.r. | n.r. |
| #17 Keller2019, U.S.A., cohort, prospective, full article. **^44^** | 2019 | 61 TaTME | 12 | 50/11 | 62.9 (10.5) | 6.18 mean,  SD 2.05 | Low/mid | 14/44/3 | 58 (95%) | 12 |
| #18 Mosquera2019, **^45^** Spain, cohort, prospective, abstract. | 2019 | 12 TaTME | n.r. | n.r. | n.r. | n.r. | low/mid | n.r. | n.r. | n.r. |
|  |  | 12 LapTME | n.r. | n.r. | n.r. | n.r. | low/mid | n.r. | n.r. | n.r. |
| #19 Leao2019, **^46^** Portugal, cohort, prospective, full article. | 2019 | 20 TaTME | 18 (4-35) | 12/8 | 60 mean, range 32-80 | all < 10 cm | low/mid | n.r. | 4 (20%) | n.r. |
| #20 Dou2019, China, cohort, retrospective, full Chinese article, only English abstract available. **^47^** | 2019 | 54 TaTME | 17.2 (12.1-30.4) | 35/54 | 57.2 median, range 26.0-77.0 | 22 <5 cm, all < 10 cm | low/mid | Majority ASA II. n.r. | 20 (37%) | n.r. |
|  |  | 53 LapTME | 17.2 (12.1-30.4) | 35/53 | 62 median, range 33.0-73.0 | 25 <5 cm, all <10 cm | low/mid | n.r. | 34 (64%) | n.r. |

| **Study ID.** | **TaTME or LapTME (n)** | **BMI (mean, SD)** | **Neoadjuvant therapy** | | **Adjuvant therapy** | | **cT (T1/T2/ T3/T4, n)** | **cN (N0/N1 /N2)** | **cM (M0/M1** | **Type of anastomosis (n)** | **Clavien Dindo Classification (I/II/III/IV/V)** |
| --- | --- | --- | --- | --- | --- | --- | --- | --- | --- | --- | --- |
|  |  |  | **N, %** | **Type, n** | **Yes/no, n** | **Type, n** |  |  |  |  |  |
| #1 Bjoern2019, Denmark, cross-sectional, retrospective, full article. **^29^** | 49 TaTME | 26.57 (3.48) | 8 (16.3%) | CRT | No | No | 0/25/23/1 | 35/6/8 | 46/3 | STE | n.r. |
|  | 36 LapTME | 25.45 (4.81) | 8 (22.2%) | CRT | No | No | 0/17/19/0 | 10/10/16 | 34/2 | STE | n.r. |
| #2 Veltcamp2018, Netherlands, cross-sectional, retrospective, full article. **^30^** | 27 TaTME | 27.6  (95%CI 25.7-29.5) | 18 (67%) | RT 16, CRT 2 | No | No | n.r. | n.r. | n.r. | ETE 23, STE 4 | IIIa-V: 3 |
|  | 27 LapTME | 26.1  (95%CI 25.1-27.3) | 22 (81%) | RT 18, CRT 4 | No | No | n.r. | n.r. | n.r. | STE | IIIa-V: 7 |
| #3 Turrado-  Rodriguez2018, Spain, cross-sectional, **^31^** retrospective, abstract. | 80 TaTME | n.r. | n.r. | n.r. | n.r. | n.r. | n.r. | n.r. | n.r. | n.r. | n.r. |
| #4 Rubinkiewicz2018, Poland, cohort, retrospective, full article. **^32^** | 25 TaTME | 24.80 median, IQR 21.94-26.47 | 24 (96%) | CRT | 13 (52%) | CT | 7/33/1/15/0 | 15/1/5 | 22/3 | Handsewn coloanal 6, circular stapled 19 | 0/1/2/0/0 |
| #5 Reali2018, U.K., cohort, prospective, abstract. **^33^** | 29 TaTME | n.r. | 8 (27.6%) | RCT | No | No | n.r. | n.r. | n.r. | n.r. | n.r. |
| #6 Mora2018, Spain, cohort, prospective, full article. **^34^** | 16 TaTME | n.r. | 7 (43.8%) | n.r. | No | No | n.r. | n.r. | n.r. | ETE | n.r. |
|  | 15 LapTME | n.r. | 11 (73.3%) | n.r. | No | No | n.r. | n.r. | n.r. | ETE | n.r. |
| #7 Foo2018, N.R., cohort, prospective, abstract. **^35^** | 23 TaTME | n.r. | n.r. | n.r. | n.r. | n.r. | n.r. | n.r. | n.r. | n.r. | n.r. |
| #8 Lelong2017, France, case-control, full article **^36^** | 34 TaTME | 24 median,  range 18.6-45.0 | 30 (88.2%) | CRT | No | No | 4/4/24/2 | 19/13 | 30/4 | J-pouch 6, Lateral colonic reconstruction 21 | n.r. |
|  | 38 LapTME | 24.2 median, range 17.7-32.7 | 35 (92.1%) | CRT | No | No | 2/5/28/3 | 18/20 | 35/3 | J-pouch 14, lateral colonic reconstruction 19 | n.r. |
| #9 Koedam2017, **^37^** Netherlands, cohort, prospective, full article. | 30 TaTME | 26.4 median,  SD 3.8 | 22 (73%) | RT 12. CRT 10 | No | No | n.r. | n.r. | n.r. | ETE 20 | III-V: 5 |
| #10 Hanke2017, Germany, cohort, prospective, abstract. **^38^** | 31 TaTME | n.r. | n.r. | n.r. | n.r. | n.r. | n.r. | n.r. | n.r. | n.r. | n.r. |
| #11 Elmore2017, Italy, cohort, prospective, abstract. **^39^** | 12 TaTME | n.r. | n.r. | n.r. | n.r. | n.r. | n.r. | n.r. | n.r. | n.r. | n.r. |
| #12 Pontallier2016, France, RCT, full article. **^40^** | 38 TaTME | 25.5 median, range 17.3-33.2 | 30 (79%) | RCT | No | No | T1-2: 8 (21%), T3-4: 30 (79%) | N0: 15 (40%), N1-2: 23 (60%) | n.r. | coloanal 18, ISR 20 | III-V: 3 |
|  | 34 LapTME | 24.8 median, range 18.3-38.3 | 30 (88%) | RCT | No | No | T1-2: 7 (21%), T3-4: 27 (79%) | N0: 12 (35%), N1-2: 22 (65%) | n.r. | Coloanal 11, ISR 23 | III-V: 3 |
| #13 Kneist2016, **^41^** Germany, cohort, prospective, full article. | 10 TaTME | 25.5 mean,  range 19.9-38.0 | 8 (80%) | CRT 6, CT 1. RT 1 | Yes | CT 8, CRT 2 | n.r. | n.r. | n.r. | STE 4, ETE 3, J-pouch 3 | n.r. |
| #14 Tuech2015, France, cross-sectional, **^42^** prospective, full article. | 56 TaTME | 27 median,  range 20-42 | 47 (84%) | CRT | n.r. | n.r. | 3/7/44/2 | n.r. | 53/3 | coloanal handsewn 46, STE 29, ETE 13 | n.r. |
| #15 Angelis2015, **^43^** France, cross-sectional, prospective, full article. | 32 TaTME | 25.19 (3.52) | 27 (84.4%) | CRT | No | No | 0/13/17/2 | 21/10/1 | n.r. | coloanal | 2/3/1/0/0 |
|  | 32 LapTME | 24.53 (3.19) | 23 (71.9%) | CRT | No | No | 0/16/13/3 | 14/15/3 | n.r. | coloanal | 2/9/1/0/0 |
| #16 Rouanet2013, **^9^** France, cohort, prospective, full article. | 21 TaTME | 26.0 median, range 21.0-32.4 | 29 (96.7%) | RT 3, CT 3, CRT 5 | No | No | 1/1/21/7 | n.r. | 27/3 | Coloanal, straight 18, J-pouch 12 | 2/9/0/1/0 |
| #17 Keller2019, U.S.A., cohort, prospective, full article. **^44^** | 61 TaTME | 26.5 (3.6) | 27 (44%) | CRT | 8 (39.3%) | n.r. | n.r. | n.r. | n.r. | ETE 26, STE 7, J-pouch 2 | 6/3/3/0/0 |
| #18 Mosquera2019, **^45^** Spain, cohort, prospective, abstract. | 12 TaTME | n.r. | n.r. | n.r. | n.r. | n.r. | n.r. | n.r. | n.r. | n.r. | n.r. |
|  | 12 LapTME | n.r. | n.r. | n.r. | n.r. | n.r. | n.r. | n.r. | n.r. | n.r. | n.r. |
| #19 Leao2019, **^46^** Portugal, cohort, prospective, full article. | 20 TaTME | 26.8 mean, range 20.4 - 36.10 | 4 (20%) | CRT | No | No | T1-3 | n.r. | All M0 | n.r. | I-II 8, >III: 2 |
| #20 Dou2019, China, cohort, retrospective, full Chinese article, only English abstract available. **^47^** | 54 TaTME | n.r. | n.r. | n.r. | n.r. | n.r. | n.r. | All Nx | All M0 | n.r. | n.r. |
|  | 53 LapTME | n.r. | n.r. | n.r. | n.r. | n.r. | n.r. | All Nx | All M0 | n.r. | n.r. |

**Table S2 Overview of quality assessment, using the Newcastle-Ottawa Scale and Cochrane Risk of Bias Tool.**

| Study code | Selection | | | | | Comparability | Outcome | | | Total score |
| --- | --- | --- | --- | --- | --- | --- | --- | --- | --- | --- |
|  | **A** | | **B** | **C** | **D** |  | **E** | **F** | **G** |  |
| Bjoern2019 ^29^ | 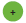 | | 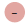 | 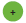 | 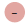 | 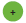 | 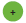 | 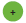 | 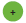 | 6 |
| Veltcamp2018 ^30^ | 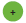 | | 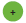 | 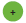 | 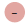 | 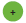 | 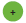 | 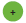 | 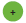 | 7 |
| TurradoRodriguez2018 ^31^ | 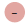 **I** | | 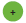 | 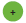 | 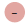 | 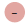 | 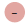 **J** | 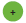 | 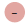 | 3 |
| Rubinkiewicz2018 ^32^ | 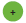 | | 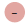 | 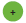 | 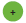 | 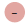 | 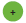 | 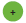 | 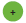 | 6 |
| Reali2018 ^33^ | 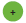 | | 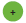 | 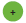 | 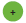 | 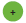 | 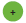 | 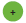 | 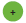 | 8 |
| Mora2018 ^34^ | 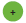 | | 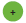 | 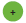 | 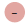 | 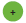 | 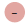 | 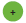 | 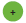 | 6 |
| Foo2018 ^35^ | 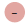 **I** | | 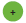 | 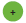 | 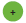 | 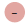 | 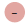 **J** | 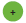 | 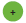 | 5 |
| Lelong2017 ^36^ | 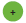 | | 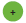 | 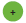 | 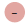 | 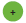 | 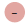 | 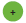 | 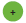 | 6 |
| Koedam2017 ^37^ | 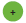 | | 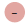 | 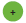 | 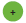 | 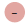 | 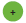 | 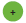 | 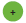 | 6 |
| Hanke2017 ^38^ | 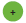 | | 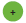 | 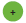 | 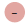 | 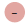 | 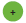 | 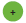 | 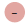 | 5 |
| Elmore2017 ^39^ | 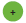 | | 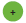 | 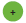 | 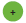 | 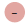 | 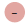 **J** | 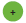 | 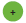 | 6 |
| Pontallier2016 ^40^ | RCT, Cochrane Risk of Bias Tool: good quality.  All domains are low risk, except unclear risk for selective reporting. | | | | | | | | | |
| Kneist2016 ^41^ | 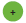 | 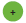 | | 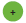 | 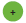 | 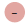 | 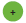 | 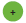 | 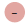 | 6 |
| Tuech2015 ^42^ | 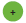 | 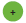 | |  |  |  |  |  |  | 6 |
| Angelis2015 ^43^ |  |  | |  |  |  |  |  |  | 6 |
| Rouanet2013 ^9^ |  |  | |  |  |  | **J** |  |  | 5 |
| Keller2019 ^44^ |  |  | |  |  |  |  |  |  | 7 |
| Mosquera2019 ^45^ |  |  | |  |  |  | **J** |  |  | 4 |
| Leao2019 ^46^ |  |  | |  |  |  |  |  |  | 6 |
| Dou2019 ^47^ |  |  | |  |  |  |  |  |  | 7 |

Abbreviations: A: Representative of exposed cohort. B: Selection of nonexposed cohort. C: Ascertainment of exposure. D: Outcome of interest absent at start of study. E: Assessment of outcome. F: Follow-up long enough for outcomes to occur. G: Adequacy of follow-up. Superscripts: ^H^ there is no need for a non-exposed cohort. Since no study had one, all of them received 1 star. ^I^ not reported. ^J^ poor descriptive outcome notation.

**Table S3 Quality of life as measured by the EQ5D.**

| **Study nr. and article ID** | **Nr. of patients** | **Time of follow-up in months (median, range) *** | **EQ5D Vas (mean, SD) *** | **EQ5D index** | **Mobility**  **(n in I/II/III)** | **Self-care**  **(n in I/II/III)** | **Activity**  **(n in I/II/III)** | **Pain/discomfort**  **(n in I/II/III)** | **Anxiety/ depression (n in I/II/III)** |
| --- | --- | --- | --- | --- | --- | --- | --- | --- | --- |
|  |  |  |  |  |  |  |  |  |  |
| **Veltcamp**  **2018 ^30^** | 27 TaTME | 20.0 (6.6-44.4) | 75.6, SD 14.41 | 88.1, SD 12.64 | 19/8/0 | 26/1/0 | 18/8/1 | 19/8/0 | 22/5/0 |
|  | 27 LapTME | 59.5 (39.7-82.0) | 79.1, SD 15.80  *p=0.400* | 92.8, SD 11.63  *p=0.159* | 22/5/0  *p=0.340* | 25/2/0 *p=1* | 22/4/0  *p=0.26* | 21/6/0 *p=0.535* | 24/3/0 *p=0.704* |
| **Koedam2017 ^37^** | 30 TaTME | Baseline | 82.5, SD 11.78 | 90.2, SD 16.87 | 28/2/0 | 30/0/0 | 26/4/0 | 23/7/0 | 23/6/1 |
|  |  | 1 month | 70, SD 17.54 | 78.2, SD 24.77 | 24/6/0 | 28/2/0 | 11/13/6 | 14/14/2 | 25/5/0 |
|  |  | 6 months | 77.5, SD 13.93 | 86, SD 16.47 | 25/5/0 | 30/0/0 | 21/7/2 | 19/11/0 | 23/7/0 |
| **Keller2019 ^44^** | 61 TaTME | Baseline | 81.8, SD 16.04 | *n.r.* | *n.r.* | *n.r.* | *n.r.* | *n.r.* | *n.r.* |
|  |  | 12 months | 76.39, SD 18.63 | *n.r.* | *n.r.* | *n.r.* | *n.r.* | *n.r.* | *n.r.* |

Abbreviations: TaTME; transanal total mesorectal excision, SD; standard deviation, VAS; Visual Analogue Scale.

***Results:*** Koedam et al. [34] and Keller et al.[37] both included a preoperative baseline measurement (baseline EQ5D-VAS scores of respectively 82.5 and 81.8). One month after surgery, Koedam et al. showed a serious deterioration of function (mean EQ5D-VAS of 70), but also signs of recovery after six to twelve months (mean EQ5D-VAS 77.5) [41]. Veltcamp et al. conducted a cohort study and found no significant difference between the TaTME and LapTME groups on the EQ5D index-, VAS- and individual subcategory scores [48].

**Table S4 Quality of life measured by EORTC QLQ-C30 and -CR29.**

| **Study nr.** | **Nr. of patients** | **Time of follow-up in months (mean, SD)*** | | | **QL** | **PF** | **RF** | **EF** | **CF** | **SF** | **FA** | **NV** |
| --- | --- | --- | --- | --- | --- | --- | --- | --- | --- | --- | --- | --- |
|  |  |  |  |  |  |  |  |  |  |  |  |  |
|  |  |  |  |  |  |  |  |  |  |  |  |  |
|  |  |  | | |  |  |  |  |  |  |  |  |
| **Bjoern2019 ^29^** | 49 TaTME | 22.69, SD 10.3 | | | 77.72 | 88.29 | 84.69 | 87.07 | 90.47 | 88.43 | 48.63 | 2.04 |
|  | 36 LapTME | 75.08, SD 17.6 | | | 79.86  p 0.63 | 89.81  p 0.69 | 85.18  p 0.77 | 93.51  p 0.041 | 95.83  p 0.07 | 93.51  p 0.27 | 44.44  p 0.92 | 1.38  p 0.98 |
| **Veltcamp**  **2018 ^30^** | 27 TaTME | 20.0 median, range 6.6-44.4 | | | 79.6 | 83.2 | 80.2 | 89.4 | 89.4 | 87.7 | 26.5 | 3.1 |
|  | 27 LapTME | 59.5 median, range 39.7-82.0 | | | 83.6  p 0.21 | 88.1  p 0.138 | 89.5  p 0.04 | 90.1  p 0.89 | 90.1  p 0.86 | 92.6  p 0.09 | 14  p 0.02 | 2.5  p 0.99 |
| **Mora2018 ^34^** | 16 TaTME | >6 months | | | 73.96 | 92.5 | 91.67 | 89.58 | 85.42 | 91.67 | 15.97 | 1.04 |
|  | 15 LapTME | >6 months | | | 72.62  p 0.87 | 86.67 p 0.27 | 79.6  p 0.26 | 77.38  p 0.03 | 83.33  p 0.78 | 86.9  p 0.60 | 22.61  p 0.46 | 0 |
| **Koedam**  **2017 ^37^** | 30 TaTME | 6 months | | | 69.94, SD 24.15 | 88.00, SD 12.77 | 76.92, SD 24.98 | 83.01, SD 20.34 | 88.00, SD 17.69 | 77.56, SD 25.79 | 21.33, SD 27.76 | 4.167, SD 9.76 |
| **Keller2019 ^44^** | 61 TaTME | Baseline | | | 75.67 | 90.18 | 85.31 | 77.34 | 91.49 | 85.05 | 22.28 | 2.74 |
|  |  | 12 months | | | 71.1  p 0.17 | 90.76 p 0.39 | 85.56  p 0.49 | 86.36  p 0.003 | 89.57  p 0.26 | 83.57  p 0.23 | 22.28  p 0.49 | 2.72  p 0.44 |
| **Meta-analysis combining p values~** | | |  |  | **p 0.85** | **p 0.55** | **p 0.53** | **p 0.15** | **p 0.80** | **p 0.84** | **p 0.65** | **p 0.44** |

Part 1/4:

Part 2/4:

| **Study nr.** | **Nr. of patients** | | **Time of follow-up in months (mean, SD)*** | | **PA** | | **DY** | | **SL** | | **AP** | | **CO** | | **DI** | | **FI** | |  |
| --- | --- | --- | --- | --- | --- | --- | --- | --- | --- | --- | --- | --- | --- | --- | --- | --- | --- | --- | --- |
|  |  | |  | |  | |  | |  | |  | |  | |  | |  | |  |
| **Bjoern2019 ^29^** | | 49 TaTME | | 22.69, SD 10.3 | | 10.20 | | 12.24 | | 18.36 | | 10.88 | | 10.88 | | 17.68 | | 1.36 | |
|  |  | 36 LapTME | | 75.08, SD 17.6 | | 8.79  p 1.00 | | 4.62  p 0.06 | | 14.81  p 0.45 | | 2.77  p 0.052 | | 2.77  p 0.55 | | 4.62  p 0.01 | | 0  p 0.22 | |
| **Veltcamp**  **2018 ^30^** | | 27 TaTME | | 20.0 median, range 6.6-44.4 | | 12.80 | | 23.50 | | 18 | | 7.40 | | 8.60 | | 16 | | 14.80 | |
|  |  | 27 LapTME | | 59.5 median, range 39.7-82.0 | | 3.70  p 0.051 | | 9.90  p 0.21 | | 14.8  p 0.39 | | 2.5  p 0.36 | | 9.9  p 0.76 | | 3.7  p 0.07 | | 2.4  p 0.03 | |
| **Mora2018 ^34^** | | 16 TaTME | | >6 months | | 5.2 | | 16.67 | | 14.58 | | 12.5 | | 22.92 | | N.R. | | N.R. | |
|  |  | 15 LapTME | | >6 months | | 13.09  p 0.24 | | 14.28  p 0.81 | | 21.42  p 0.43 | | 2.38  p 0.19 | | 33.33  p 0.38 | | N.R. | | N.R. | |
| **Koedam**  **2017 ^37^** | | 30 TaTME | | 6 months | | 13.58, SD 20.69 | | 11.91, SD 28.99 | | 10.71, SD 25.75 | | 7.14, SD 22.87 | | 14.10, SD 26.95 | | 14.67, SD 27.35 | | 8.97, SD 22.23 | |
| **Keller2019 ^44^** | | 61 TaTME | | Baseline | | 12.87 | | 8.13 | | 19.07 | | 8.16 | | 5.98 | | 25.1 | | 12.57 | |
|  |  |  |  | 12 months | | 10.89  p 0.43 | | 5.97  p 0.27 | | 16.58  p 0.42 | | 7.05  p 0.46 | | 7.61  p 0.38 | | 22.85  p 0.43 | | 11.43  p 0.50 | |
| **Meta-analysis combining p-values~** | | |  |  | | **p 0.46** | | **p 0.97** | | **p 0.67** | | **p 1.0** | | **p 0.44** | | **p 1.0** | | **p 0.99** | |

Part 3/4:

| **Study nr.** | **Nr. of patients** | | **Time of follow-up in months (mean, SD)*** | **UF** | **BMS** | **BI** | **UI** | **DY** | **AP** | **BP** | **BF** | **DM** | **HL** | **TT** |
| --- | --- | --- | --- | --- | --- | --- | --- | --- | --- | --- | --- | --- | --- | --- |
|  |  |  |  |  |  |  |  |  |  |  |  |  |  |  |
|  |  |  |  |  |  |  |  |  |  |  |  |  |  |  |
|  |  | |  |  |  |  |  |  |  |  |  |  |  |  |
| **Bjoern2019 ^29^** | 49 TaTME | | 22.69, SD 10.3 | 89.34 | 4.76 | 89.34 | 2.04 | 2.04 | 8.16 | 14.28 | 17.68 | 18.36 | 2.72 | 4.16 |
|  | 36 LapTME | | 75.08, SD 17.6 | 19.44  p 0.65 | 0.92  p 0.18 | 88.58  p 0.65 | 3.70  p 0.67 | 1.85 p 0.77 | 11.11 p 0.33 | 2.77  p 0.01 | 12.96 p 0.36 | 10.18  p 0.39 | 1.85  p 0.89 | 0  p 0.047 |
| **Veltcamp**  **2018 ^30^** | 27 TaTME | | 20.0 median, range 6.6-44.4 | 38.9 | 3.7 | 88.4 | 7.4 | 2.5 | 10.3 | 24.7 | 14.8 | 29.8 | 9.9 | 17.3 |
|  | 27 LapTME | | 59.5 median, range 39.7-82.0 | 28.4  p 0.10 | 3.7  p 1.0 | 90.9  p 0.33 | 9.9  p 0.89 | 1.2  p 0.56 | 7.4  p 0.64 | 12.3  p 0.11 | 14.8  p 1.0 | 8.6  p 0.16 | 0  p 0.01 | 6.2  p 0.08 |
| **Mora2018 ^34^** | 16 TaTME | | >6 months | n.r. | n.r. | 90.97 | 8.33 | 4.44 | 11.11 | 18.75 | 14.58 | n.r. | n.r. | n.r. |
|  | 15 LapTME | | >6 months | n.r. | n.r. | 85.19  p 0.43 | 8.89  p 0.92 | 6.67  p 0.77 | 28.89  p 0.04 | 28.89  p 0.34 | 37.78  p 0.04 | n.r. | n.r. | n.r. |
| **Koedam**  **2017 ^37^** | 30 TaTME | | 6 months | 23.46, SD 16.83 | 7.41, SD 11.63 | 81.89, SD 19.53 | 7.41, SD 16.88 | 2.46, SD 8.89 | 3.70, SD 10.68 | 28.39, SD 25.66 | 6.17, SD 13.19 | 14.81, SD 25.04 | 4.94, SD 15.20 | 4.94, SD 15.20 |
| **Keller2019 ^44^** | 61 TaTME | | Baseline | n.r. | n.r. | 88.59 | n.r. | n.r. | 9.82 | n.r. | 17.93 | 9.75 | 3.79 | 9.25 |
|  |  |  | 12 months | n.r. | n.r. | 87.87  p 0.37 | n.r. | n.r. | 8.48  p 0.28 | n.r. | 19.02  p 0.29 | 10.34  p 0.48 | 3.82  p 0.33 | 6.52  p 0.36 |
| **Meta-analysis combining p values~** | |  |  | **p 0.59** | **n.p.** | **p 0.46** | **p 0.49** | **p 0.74** | **p 0.06** | **p 0.72** | **p 0.09** | **p 0.06** | **p 0.88** | **p 1.0** |

Part 4/4:

| **Study nr.** | **Nr. of patients** | **Time of follow-up in months (mean, SD)*** | **AN** | **WE** | **FL** | **FI** | **SS** | **SF** | **EDP** | **MSF** | **MI** | **FSF** | **FD** |
| --- | --- | --- | --- | --- | --- | --- | --- | --- | --- | --- | --- | --- | --- |
|  |  |  |  |  |  |  |  |  |  |  |  |  |  |
|  |  |  |  |  |  |  |  |  |  |  |  |  |  |
|  |  |  |  |  |  |  |  |  |  |  |  |  |  |
| **Bjoern2019 ^29^** | 49 TaTME | 22,69, SD 10,3 | 79.59 | 84.35 | 32.65 | 20.40 | 14.96 | 19.79 | 10.20 | 50.45 | 50.45 | 5.55 | 0 |
|  | 36 LapTME | 75,08, SD 17,6 | 81.48  p 0.95 | 86.11  p 0.60 | 26.85  p 0.39 | 13.88  p 0.13 | 7.4  p 0.13 | 17.22 p 0.44 | 8.33  p 0.32 | 50  p 0.96 | 48.33  p 0.77 | 20.83  p 0.053 | 2.08  p 0.80 |
| **Veltcamp**  **2018 ^30^** | 27 TaTME | 20,0 median, range 6,6-44,4 | 74.4 | 87.2 | 41 | 33.3 | 26.9 | 36.5 | 38.5 | 68.9 | 41 | 83.3 | 7.4 |
|  | 27 LapTME | 59,5 median, range 39,7-82,0 | 75.3  p 0.71 | 84.1  p 0.49 | 39.7  p 0.98 | 16.7  p 0.03 | 7.7  p 0.02 | 30.7  p 0.56 | 28.2 p 0.18 | 63.3  p 0.56 | 51  p 0.48 | 73.3  p 0.66 | 8.3  p 0.91 |
| **Mora2018 ^34^** | 16 TaTME | >6 months | 72.92 | 66.67 | 51.28 | 28.2 | 2.51 | 25.64 | n.r. | 53.33 | 51.85 | 86.33 | 8.33 |
|  | 15 LapTME | >6 months | 64.44  p 0.49 | 77.78  p 0.36 | 47.22  p 0.79 | 33.3  p 0.69 | 13.89  p 0.53 | 36.1  p 0.33 | n.r. | 44.44  p 0.63 | 66.67  p 0.47 | 88.89  p 0.72 | 13.33  p 0.76 |
| **Koedam**  **2017 ^37^** | 30 TaTME | 6 months | 24.69, SD 19.81 | 7.41, SD 14.12 | 22.22, SD 26.15 | 37.04, SD 31.12 | 33.33, SD 26.15 | 36.42, SD 26.16 | 43.59, SD 33.69 | 23.33, SD 19.04 | 51.11, SD 43.40 | 16.67, SD 18.26 | 20.00, SD 18.26 |
| **Keller2019 ^44^** | 61 TaTME | Baseline | 31.11 | n.r. | n.r. | 21.16 | 16.46 | n.r. | 22.38 | 45.83 | 21.26 | n.r. | 21.26 |
|  |  | 12 months | 19.46  p 0.00 | n.r. | n.r. | 26.42  p 0.11 | 22.07  p 0.11 | n.r. | 23.66  p 0.17 | 48.26  p 0.29 | 27.25  p 0.06 | n.r. | 27.25  p 0.06 |
| **Meta-analysis**  **combining p values~** | | | **p 0.54** | **p 0.69** | **p 0.84** | **p 0.89** | **p 0.83** | **p 0.57** | **p 0.97** | **p 0.39** | **p 0.35** | **p 0.79** | **p 0.50** |

Abbreviations: # expressed as p-value with chi-square test. * if no mean available; the used descriptive value is named. ~: only possible if a comparison between TaTME and LapTME is made. ^ Overall scores are reported (no subgroups with no stoma/with stoma). QL: global health status, PF: physical functioning, RF: role functioning, EF: emotional functioning, CF: cognitive functioning, SF: social functioning, FA: fatigue, NV: nausea and vomiting, PA: pain, DY: dyspnoea, SL: insomnia, AP: appetite loss, CO: constipation, DI: diarrhoea, FI: financial problems. UF; urinary frequency, BMS; Blood and mucus in stool, BI; body image, UI: urinary incontinence, DY: dysuria, AP: abdominal pain, BP: buttock pain, BF: bloated feeling, DM: dry mouth, HL: hair loss, TT: trouble with taste, AN: anxiety, WE: weight, FL: flatulence, FI: faecal incontinence, SS: sore skin around anus, SF: stool frequency, EDP: embarrassed by defecation pattern, MSF: Male sexual functioning, MI: male impotence, FSF: female sexual functioning, FD: female dyspareunia. N.r.: not reported. N.p.: not possible.

Abbreviations: # expressed as p-value with chi-square test. * if no mean available; the used descriptive value is named. ~: only possible if a comparison between TaTME and LapTME is made. ^ Overall scores are reported (no subgroups with no stoma/with stoma). QL: global health status, PF: physical functioning, RF: role functioning, EF: emotional functioning, CF: cognitive functioning, SF: social functioning, FA: fatigue, NV: nausea and vomiting, PA: pain, DY: dyspnoea, SL: insomnia, AP: appetite loss, CO: constipation, DI: diarrhoea, FI: financial problems. UF; urinary frequency, BMS; Blood and mucus in stool, BI; body image, UI: urinary incontinence, DY: dysuria, AP: abdominal pain, BP: buttock pain, BF: bloated feeling, DM: dry mouth, HL: hair loss, TT: trouble with taste, AN: anxiety, WE: weight, FL: flatulence, FI: faecal incontinence, SS: sore skin around anus, SF: stool frequency, EDP: embarrassed by defecation pattern, MSF: Male sexual functioning, MI: male impotence, FSF: female sexual functioning, FD: female dyspareunia. N.r.: not reported. N.p.: not possible.
